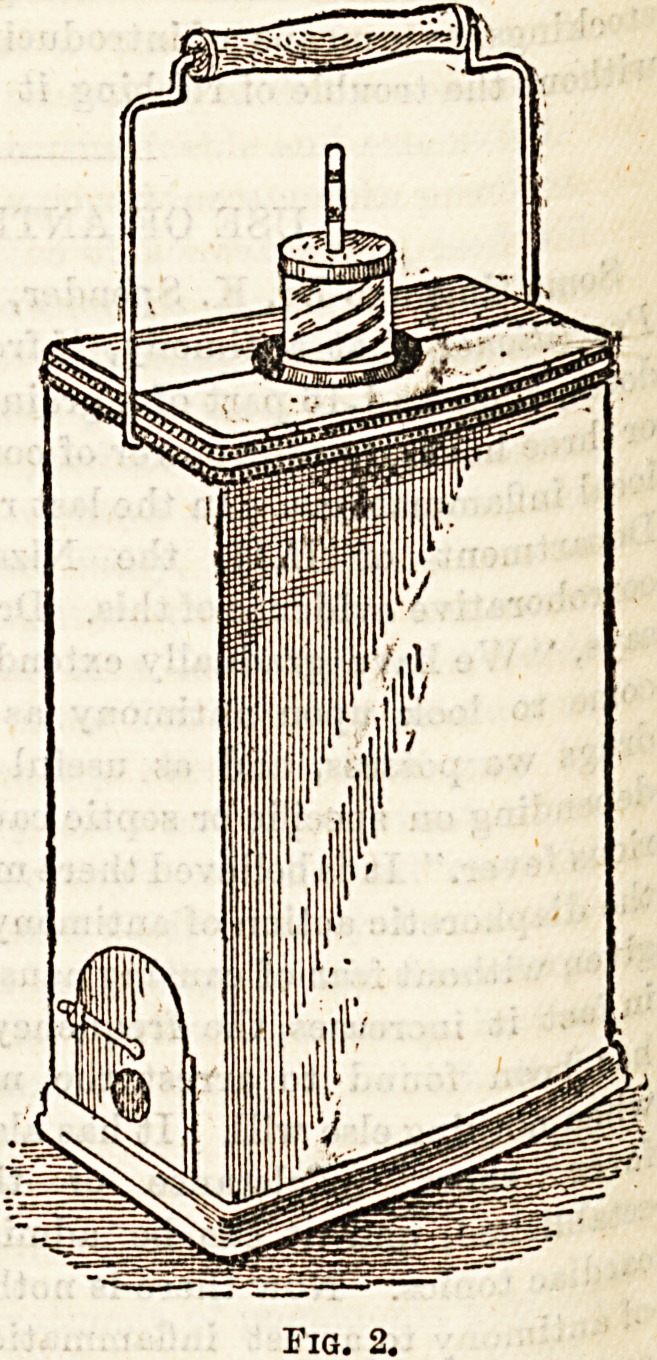# New Drugs, Appliances, and Things Medical

**Published:** 1890-03-01

**Authors:** 


### NEW DRUGS, APPLIANCES, AND THINGS MEDICAL.

[All preparations, appliances, novelties, etc., of which a notice is desired, should be sent to The Editor, The Lodge, Porchester Square, W.]

### THE LACTOTHERME.

This is an infant's portable milk stove, patented in England and abroad, the sole agent for which is Mr. Wm. Toogood. The object of the Lactotherme is to heat milk and maintain it at a temperature of 98 deg. Fahrenheit, so benefitting the health of infants by always securing that the food shall be given at a proper temperature. It is also suitable for the use of invalids, being specially adapted to the maintenance of a uniform temperature in fluid food of all kinds. This uniformity of temperature is maintained by the Lactotherme for a period of six or seven hours, and when once lighted needs no further attention. It will be seen from the diagrams that the apparatus consists of two compartments, the upper which is lined with felt, into which the food receptacle is inserted; and the lower, consisting of the heating chamber, which contains an open grid for the reception of specially-prepared carbon blocks, or which can be utilised to support a tin lamp containing oil and a floating wick.

Fig. 1 shows the lactotherme in section with the vessel containing food, and the thermometer to show that the desired temperature is maintained. Fig 2. shows the apparatus closed. It will be seen from Fig. 1, that the pad of felt upon which the bottle rests can be removed by the wire thread shown in the drawing when the food is placed in the apparatus cold. So soon as it has reached the desired temperature, if the little pad is replaced and the bottle inserted, 98 degrees of heat will be maintained for several hours. This apparatus is very simple, and may be used by anybody of ordinary intelligence without danger or difficulty. It will prove a boon to travellers, being excellently adapted for this purpose. We should recommend the

Fig. 1.

Fig. 2.

use of No. 2 pattern, polished brass, in preference to any other. We would further suggest that the apparatus would be improved by the addition of a thermometer of better quality and registering more than 98 deg. Users should also be cautioned against placing boiling water in the bottle or vessel used for food, as often people do not know what constitutes boiling point, and very many thermometers are liable to be broken by plunging them into boiling water. Night lights cannot be used for this apparatus, and it is indispensable that nothing should be added to the milk except pure water.

### THE ELECTRIC CYSTOSCOPE.

The February number of the *Edinburgh Medical Journal* contains an interesting article from the pen of Mr. David Wallace on the "Electric Cystoscope." After a *résumé* of the history of the invention, Mr. Wallace mentions two forms of instrument, an anterior and a posterior. Each is shaped like a sound, and consists of three parts, the beak, the shaft, and the eye-piece. Running from the eye-piece to the beak there are two hollow tubes, an inner and an outer. The former serves to connect the electrodes of a battery—the source of electro-motive force—with the lamp the source of light. The beak is a hollow cap, which has a window of rock-crystal, and contains a small Swan incandescent lamp, from which the light for illumination is emitted. In the anterior instrument, at the concavity where the shaft and beak join, there is a prism which refracts the rays of light from the object looked at on to the end of a telescope, which passes from the eye-piece down to the junction of the shaft and beak. In the posterior instrument no prism is necessary, but merely a plate of glass, as the object to be observed is in a line with the telescope and the observer's eye. The upper three-fourths of the bladder may be examined with the anterior cystoscope, the lower fourth with the posterior. Mr. Wallace considers the cystoscope will prove a most valuable aid in diagnosis of bladder diseases.

## Figures and Tables

**Fig. 1. f1:**
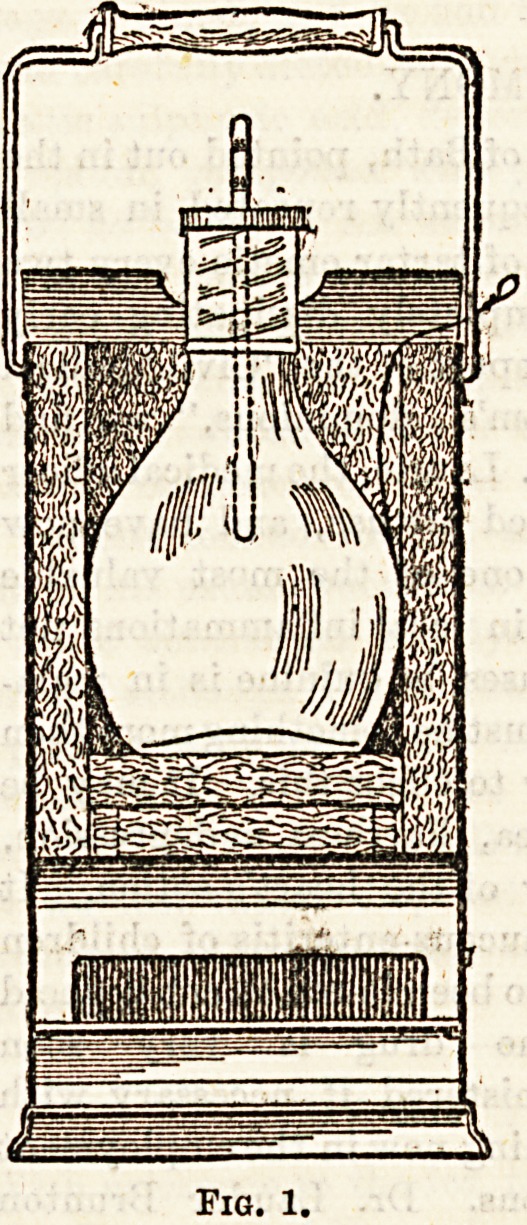


**Fig. 2. f2:**